# A Novel microRNA of Japanese Flounder Regulates Antimicrobial Immunity Involving a Bacteria-Binding CSF3

**DOI:** 10.3389/fimmu.2021.723401

**Published:** 2021-08-19

**Authors:** Wenrui Li, Xiaolu Guan, Bin Sun, Li Sun

**Affiliations:** ^1^CAS Key Laboratory of Experimental Marine Biology, Institute of Oceanology, Center for Ocean Mega-Science, Chinese Academy of Sciences, Beijing, China; ^2^Laboratory for Marine Biology and Biotechnology, Pilot National Laboratory for Marine Science and Technology (Qingdao), Qingdao, China; ^3^College of Marine Science, University of Chinese Academy of Sciences, Beijing, China

**Keywords:** *Paralichthys olivaceus*, fish microRNA, granulocyte colony stimulating factor 3, bacterial and viral pathogens, antimicrobial immune defense

## Abstract

MicroRNAs (miRNAs) are small non-coding RNAs that regulate diverse biological processes including immunity. In a previous high-throughput RNA sequencing study, a novel miRNA, pol-miR-novel_642, was identified from Japanese flounder (*Paralichthys olivaceus*), a farmed fish species with important economic value. In this study, we investigated the regulatory mechanism and the function of pol-miR-novel_642 and its target gene. We found that pol-miR-novel_642 targeted, in a sequence-specific manner, a flounder gene encoding an uncharacterized protein that is a structural homologue of murine granulocyte colony stimulating factor 3 (CSF3). The expression of pol-miR-novel_642 and its target gene (named PoCSF3-1) was regulated, in different manners, by the bacterial pathogen *Edwardsiella tarda* and the viral pathogen megalocytivirus. Overexpression of pol-miR-novel_642 or interference with PoCSF3-1 expression in flounder cells strongly potentiated *E. tarda* infection. Consistently, *in vivo* knockdown of PoCSF3-1 enhanced bacterial dissemination in flounder tissues but blocked viral replication, whereas *in vivo* overexpression of PoCSF3-1 inhibited bacterial dissemination and facilitated viral infection. Overexpression/knockdown of PoCSF3-1 and pol-miR-novel_642 also affected the activation of autophagy. Recombinant PoCSF3-1 (rPoCSF3-1) interacted with and inhibited the growth of Gram-negative bacteria in a manner relying on a PoCSF3-1-characteristic structural motif that is absent in mouse CSF3. rPoCSF3-1 also regulated the proliferation, inflammatory response, and immune defense of flounder head kidney leukocytes in a structure-dependent fashion. Together, these results reveal the function of a novel miRNA-CSF3 regulatory system of flounder, and add new insights into the role and mechanism of fish miRNA and CSF3 in antimicrobial immunity.

## Introduction

MicroRNAs (miRNAs) are a type of small noncoding RNAs that negatively regulate the expression of protein-coding genes (1–3). MiRNAs are present widely in animals, plants, and viruses. In animals, miRNAs most often inhibit their target genes by blocking mRNA translation or decreasing mRNA stability *via* interaction with the 3’ untranslated regions (3’UTRs) of the target genes (4, 5). However, recent studies showed that some miRNAs interact with the coding regions of the target genes to modulate gene expression (1, 6–8). MiRNAs regulate diverse biological processes and signal pathways involved in infection and immunity (9–13). As a result, miRNAs play an important role in pathogen-induced immune response and are known to influence the outcome of host-pathogen interaction in mammals and teleost (14–18). In fish, miRNAs associated with bacterial and viral infection have been identified in various species, and some of the miRNAs were found to function by targeting specific immune genes such as IL-1 receptor-associated kinase 4 and signal transducer and activator of transcription (STAT) 3 (19–23).

Colony stimulating factor (CSF) superfamily proteins are hematopoietic growth factors that regulate proliferation, differentiation, and survival of lineage-specific myeloid cells (24). In mammals, the family had three canonical members, namely macrophage-colony stimulating factor (M-CSF or CSF1), granulocyte/macrophage-colony stimulating factor (GM-CSF or CSF2), and granulocyte-colony stimulating factor (G-CSF or CSF3) (25). CSF3 is a low molecular weight glycoprotein produced by monocytes, fibroblasts, and endothelial cells (26). It stimulates granulocyte production and regulates the migration and antimicrobial activity of neutrophils (27, 28). CSF3 also plays a role in regulating host immune response to pathogens such as *Listeria monocytogenes* and *Candida albicans* (28, 29). In teleost, CSF3-like genes have been identified in a number of species, including Japanese flounder (*Paralichthys olivaceus*), fugu (*Takifugu rubipes*), green-spotted pufferfish (*Tetraodon nigroviridis*), black rockfish (*Sebastes schlegelii*), zebrafish (*Danio rerio*), rock bream (*Oplegnathus fasciatus*), and large yellow croaker (*Larimichthys crocea*) (30–34). Two paralogs of CSF3 have been reported to exist in some fish, including common carp, rock bream, and zebrafish, likely as a result of genome duplication (33, 35–37). Several reports showed that some fish CSF3 members are involved in pathogen infection and able to promote leukocyte proliferation, phagocytosis, or migration (33, 34, 36–39),

*Edwardsiella tarda* is a Gram-negative bacterium and a severe pathogen to many farmed fish including Japanese flounder, a type of flat fish farmed worldwide. Flounder miRNAs involving in *E. tarda* infection have been reported previously (10, 11, 17, 19, 40–44). In a previous study, we identified 96 flounder miRNAs that exhibited differential expression during *E. tarda* infection (11). One of these miRNAs is Pol-miR-novel_642, a novel miRNA with unknown function. In the present study, we examined the function of pol-miR-novel_642 and its target gene, an uncharacterized paralog of CSF3 (named PoCSF3-1). Our results showed that pol-miR-novel_642 regulates host immune response and pathogen infection in a manner that involves PoCSF3-1, and that PoCSF3-1 possesses the properties of both a cytokine and a bacterial binding protein in a structure-dependent manner.

## Materials and Methods

### Animals

Healthy Japanese flounder (average weight 20.2 g) were purchased from a commercial fish farm in Shandong Province, China, and temporarily kept at 22°C in aerated seawater. Before tissue collection, the fish were euthanized with tricaine methanesulfonate solution (Sigma, USA) as reported previously (45).

### Cell Lines

FG-9307 cells (46), a Japanese flounder gill cell line, were cultured at 24°C in L-15 medium (Thermo Scientific HyClone, USA) supplemented with 10% FBS, 100 units/mL penicillin, and 100 μg/mL streptomycin as reported previously (19). HEK293T cells were cultured at 37°C in DMEM medium (Invitrogen, Grand Island, USA) supplemented with 10% FBS, 100 units/mL penicillin, and 100 μg/mL streptomycin under humidified condition with 5% CO_2_ as previously reported (19). FG-9307 cells were used in this study because it is derived from flounder. HEK293T cells were used in luciferase reporter assay, because efficient transfection can be achieved with this cell line.

### MiRNA Mimic, Inhibitor, and Small Interfering RNA (siRNA)

The mimics of pol-miR-novel_642, pol-miR-novel_642M (with the seed sequence of 5’-AGGCUGC-3’ mutated to 5’-UCCGACG-3’) and their negative control (miR-C) were synthesized by GenePharma (Shanghai, China). Pol-miR-novel_642 inhibitor and its negative control (miR inhibitor-C) were designed and synthesized by the same company. The PoCSF3-1 specific siRNA (siPoCSF3-1) and its negative control (siC) were designed and synthesized by Ribobio (Guangzhou, China).

### Quantitative Real-Time Reverse Transcription-PCR (qRT-PCR)

To examine pol-miR-novel_642 expression in flounder during pathogen infection, *E. tarda* was cultured in LB medium at 28°C to mid-logarithmic phase and resuspended in PBS to 10^8^ CFU/mL as reported previously (47). The fish viral pathogen megalocytivirus RBIV-C1 (48) was resuspended in PBS to 5×10^5^ copies/mL. Flounder (as described above) were randomly divided into three groups and injected intraperitoneally with 100 μL suspension of *E. tarda*, megalocytivirus, or PBS. Kidney was taken aseptically from the fish at 12 h, 24 h, and 48 h post-bacterial infection and at 2 d, 4 d, 6 d, 8 d, and 10 d post-viral infection. To examine pol-miR-novel_642 expression, miRNA was isolated with miRNAiso plus kit (TaKaRa, China). The miRNA-specific reverse transcription was performed using miRNA first-strand cDNA synthesis kit (Vazyme, China) with specific stem-loop reverse transcription (RT) primer (5’-GTCGTATCCAGTGCAGGGTCCGAGGTATTCGCACTGGA TACGACGCTCAG-3’). MiRNA level was validated by qRT-PCR with SYBR ExScript qRT-PCR Kit (TaKaRa, Japan) and a QuantStudio 3 Real-time PCR system (Applied Biosystems, USA) as reported previously (19). 5s rRNA was used as an internal control. The primers used in the PCR are listed in **Table S1**.

To examine PoCSF3-1 expression in flounder during pathogen infection, the fish were injected intraperitoneally with *E. tarda* or megalocytivirus as mentioned above. Total RNA was isolated from kidney with TRIzol reagent (Invitrogen, USA). cDNA synthesis was performed with First Strand cDNA Synthesis Kit (ToYoBo, Japan) according to the manufacturer’s protocol. PoCSF3-1 expression was determined by qRT-PCR with primers PoCSF3-1-F and PoCSF3-1-R (**Table S1**). 18s rRNA and elongation factor-1-α (EF1A) were used as internal references for bacterial and viral infections, respectively, according previous reports (49, 50).

### Plasmid Construction

To construct the plasmid pPoCSF3-1-Report, the coding sequence of PoCSF3-1 was amplified by PCR with primers PoCSF3-1-CDS-F1 and PoCSF3-1-CDS-R1 (**Table S1**). The PCR product was inserted into pmirGLO (Promega, USA) at between the Nhe I and Sal I sites as reported previously (41). To construct the plasmid pPoCSF3-1si, which expresses a PoCSF3-1 specific siRNA, a siRNA targeting PoCSF3-1 was chemically synthesized (TsingKe, China) and inserted into pRNAT-CMV3.1 (GenScript, USA) at between the BamH I and Alf II sites as reported previously (41). pPoCSF3-1siC, which expresses a nonspecific siRNA, was constructed similarly. To construct the plasmid pPoCSF3-1, which expresses PoCSF3-1, the coding sequence of PoCSF3-1 was amplified by PCR with the primers PoCSF3-1-CDS-F2 and PoCSF3-1-CDS-R2 (**Table S1**), and the PCR product was inserted into the EcoR V site of pCN3 as reported previously (51). To construct the plasmid pETPoCSF3-1, which expresses His-tagged PoCSF3-1, the coding sequence of PoCSF3-1 was amplified by PCR with the primers PoCSF3-1-CDS-F3 and PoCSF3-1-CDS-R3 (**Table S1**), and the PCR product was inserted into pET28a (Novagen, USA) at between the EcoR I and Hind III sites. To construct the plasmid pETPoCSF3-1M, which expresses the mutant PoCSF3-1 (PoCSF3-1M) with ^36^ILL^39^ to ^36^SLE^39^ substitution, the coding sequence of the mutant was amplified by PCR with the primers PoCSF3-1-Mut-F and PoCSF3-1-Mut-R (**Table S1**) using Fast Mutagenesis System (TransGen Biotech, China), and the product was inserted into pET28a as above. All plasmids were verified by DNA sequencing.

### Regulation of PoCSF3-1 by pol-miR-novel-642

Luciferase reporter assay was performed as reported previously (41). Briefly, HEK293T cells were distributed in 24-well plates (about 80% confluence) and transfected with pPoCSF3-1-Report (control), pPoCSF3-1-Report plus pol-miR-novel_642 mimic, pPoCSF3-1-Report plus miR-C, or pPoCSF3-1-Report plus pol-miR-novel_642M. The transfection was carried out with Lipofectamine 3000™ reagent (Invitrogen, USA) at a 1:1 ratio of plasmid: transfection reagent for 24 h. The cells were collected, and luciferase activity was assayed using the Dual Luciferase Reporter Assay Kit (Vazyme, Nanjing, China) according to the manufacturer’s protocol. To examine the regulation of PoCSF3-1 by pol-miR-novel-642 in flounder cells, FG-9307 cells were distributed in 6-well plates (about 80% confluence) and transfected with or without pol-miR-novel_642 mimic or miR-C for 24 h. qRT-PCR was performed to determine PoCSF3-1 mRNA expression with primers as described above. Western blot was performed to determine PoCSF3-1 protein expression as reported previously (41). Briefly, the transfected cells were lysed with RIPA lysis buffer (Beyotime Biotechnology, China) on ice for 30 min. The cell lysates were mixed with 5× sodiumdodecylsulfate-polyacrylamide gelelectrophoresis (SDS-PAGE) loading buffer, boiled for 10 min, and separated by SDS-PAGE. After electrophoresis, the proteins were transferred to a nitrocellulose blotting membrane (GE healthcare, Germany). The membrane was blocked with 5% skim milk, followed by incubation with anti-PoCSF3-1 antibody (Sangon Biotech, China) or anti-β-actin antibody (ABclonal, China) at 4°C for overnight. The membrane was incubated with HRP-conjuncted anti-rabbit antibody (Abcam, UK) for 1 h at room temperature. The membrane was then incubated with ECL substrate (Beyotime Biotechnology, China) and visualized with GelDoc XR System (Bio-Rad, USA).

### Sequence Analysis

The amino acid sequence of PoCSF3-1 (accession no. XP_019938527) was analyzed with the BLAST program at the National Center for Biotechnology Information (NCBI). The amino acid sequence of *Mus musculus* CSF3 (accession no. EDL16160.1) is available from NCBI. Multiple sequence alignment was created with DNAMAN. The potential three-dimensional structure was created using the SWISS-MODEL prediction algorithm.

### Overexpression and Knockdown of pol-miR-novel_642/PoCSF3-1

For pol-miR-novel_642 overexpression in FG-9307 cells, the cells were transfected with pol-miR-novel_642 mimic or miR-C as above. For PoCSF3-1 knockdown in FG-9307 cells, the cells were transfected with siPoCSF3-1 or siC as above. To examine the effect of pol-miR-novel_642 overexpression or PoCSF3-1 knockdown on *E. tarda* infection, the above transfected FG-9307 cells were infected with *E. tarda* at a MOI of 20. At 2 h, 4 h, and 8 h post infection, intracellular bacterial number was determined by plate count as reported previously (19). For PoCSF3-1 knockdown and overexpression in flounder, flounder were injected intramuscularly with pPoCSF3-1si, pPoCSF3-1siC, pPoCSF3-1 or pCN3 at the dose of 1 μg plasmid/1 g fish. The control group was similarly injected with the same volume of PBS. Kidney, spleen and liver were harvested from the fish at 7 d post-injection. PoCSF3-1 knockdown and overexpression were verified by qRT-PCR as described above (**Figure S1**). To examine the effect of PoCSF3-1 knockdown and overexpression on pathogen infection, flounder were administered with pPoCSF3-1si, pPoCSF3-1siC, pPoCSF3-1, pCN3 or PBS for 7 days. The fish were infected with *E. tarda* or megalocytivirus as above. At 12 h, 24 h, and 48 h post *E. tarda* infection, the numbers of *E. tarda* in kidney, spleen and liver were determined by plate count as previously reported (41). At 6 d and 8 d post-megalocytivirus infection, the viral loads in kidney were determined by absolute quantitative real time RT-PCR as reported previously (16).

### Effects of PoCSF3-1 and pol-miR-novel_642 on Autophagy

To examine the effect of PoCSF3-1 knockdown and overexpression on autophagy gene expression in flounder, flounder were administered with pPoCSF3-1si, pPoCSF3-1siC, pPoCSF3-1, pCN3 or PBS. At 7 d post-plasmid administration, the expression of ATG5, AKT, mTOR, and beclin-1 in kidney, spleen and liver was examined by qRT-PCR. To examine the effect of pol-miR-novel_642 on autophagy in flounder cells, FG-9307 cells were transfected with pol-miR-novel_642 inhibitor or miR inhibitor-C for 24 h. Beclin-1 and LC3 proteins were detected by Western blot as described above.

### Purification and Recombinant Proteins

Recombinant proteins were purified as reported previously (19). Briefly, *Escherichia coli* BL21 (DE3) (TransGen Biotech, China) was transformed with pETPoCSF3-1, pETPoCSF3-1M, or pET32a (which expresses His-tagged Trx). The transformants were cultured in LB medium at 37°C to OD_600_ of 0.5. Isopropyl-β-D-thiogalactopyranoside was added to the medium to a final concentration of 0.04 mM. The culture was continued at 16°C for 16 h, and the cells were harvested by centrifugation. rPoCSF3-1, rPoCSF3-1M, and rTrx were purified using Ni-NTA agarose (QIAGEN, USA).

### Binding of rPoCSF3-1 to Bacteria and Its Effect on Bacterial Growth

The bacteria used in this study, i.e., E. tarda, Vibrio anguillarum, Vibrio harveyi, Pseudomonas fluorescens, E. coli, Bacillus subtilis, Micrococcus luteus, Streptococcus iniae, and Staphylococcus aureus, have been reported previously (52). Protein binding to bacteria was determined by enzyme-linked immunosorbent assay (ELISA) as reported previously (52). Briefly, the bacteria were resuspended in coating buffer (15 mM Na_2_CO_3_, 35 mM NaHCO_3_, pH 9.6) to 10^8^ CFU/mL. Each of the bacterial suspension was added to 96-well plates (100 μL/well). The plates were incubated at 4°C for overnight and then blocked with 5% skim milk powder at 4°C for 12 h. The plates were washed extensively with PBS containing 0.1% Tween-20 (PBST). Different concentrations of rPoCSF3-1 and rTrx were added to the plates (100 μL/well). The plates were incubated at 22°C for 2 h, followed by extensive washing with PBST. Horseradish peroxidase (HRP)-conjugated mouse anti-His antibody (ABclonal, China) was added to the plates. The plates were incubated at 37°C for 1 h, followed by extensive washing with PBST. Color development was performed using the TMB Kit (Tiangen, China). The plates were read at 450 nm, and binding index was defined as the fold increase of the reading of rPoCSF3-1-treated bacteria compared to that of the PBS-treated bacteria. To determine the effect of rPoCSF3-1 on bacterial growth, E. tarda and V. harveyi were suspended in LB medium to 10^5^ CFU/mL in 96-well plates (100 μL/well). rPoCSF3-1 or rTrx was added to each well to the final concentration of 5 μM. The same volume of PBS was added to the control sample. The plates were incubated at 28°C, and bacterial growth was determined by measuring OD_600_ at every hour. rPoCSF3-1 binding to lipid A was performed as above, except that the bacteria were replaced by E. coli lipid A (Sigma, USA). Binding of rPoCSF3-1M to lipid A and bacteria were performed as above.

### Effects of rPoCSF3-1 and rPoCSF3-1M on Head Kidney Leukocytes (HKLs)

To examine the effect of rPoCSF3-1 and rPoCSF3-1M on HKLs proliferation, flounder HKLs were isolated using density Percoll gradient centrifugation (53). The cells were distributed in a 96-well plate (1×10^6^ cells/well) and incubated with 5 μM rPoCSF3-1, rPoCSF3-1M, or rTrx at 22°C for 12 h, 24 h, and 48 h. The control cells were incubated with PBS. Cell proliferation was then determined using the cell counting kit-8 (Vazyme Biotech, Nanjing, China) according to the manufacturer’s instruction. To examine the effect of rPoCSF3-1 on gene expression, HKLs (1×10^6^ cells/mL) were incubated with rPoCSF3-1 (5 μM), rPoCSF3-1M (5 μM), rTrx (5 μM), or PBS at 22°C for 12 h, 24 h, and 48 h. qRT-PCR was performed to examine the expression of TNF-α, IL-1β, IL-6, IL-8, janus kinase (JAK)2, signal transducer and activator of transcription (STAT)1, STAT3, STAT4, and STAT6. The primers used in the PCR are listed in **Table S1**. To examine the effect of rPoCSF3-1 and rPoCSF3-1M on bacterial infection, HKLs (1×10^6^ cells/mL) were incubated with rPoCSF3-1 (5 μM), rPoCSF3-1M (5 μM), rTrx (5 μM), or PBS at 22°C for 2 h, followed by infection with *E. tarda* (1×10^6^ CFU/mL). The cells were then incubated at 22°C for 2 h. Extracellular bacteria were killed by adding gentamicin (200 μg/mL) to the culture. The cells were then incubated at 22°C for 2 h. After incubation, the cells were washed two times with PBS and cultured in L-15 medium containing 30 μg/mL gentamicin for 2 h or 4 h. The cells were collected by centrifugation at 600 g for 10 min and washed two times with PBS. The cells were lysed with 1% Triton X-100, and the lysate was diluted and plated on LB agar plates. The plates were incubated at 28°C for 48 h, and the colonies emerged on the plates were counted.

### Effects of rPoCSF3-1 and rPoCSF3-1M on Bacterial Survival in Host Serum

*E. tarda* (10^4^ CFU) were incubated with rPoCSF3-1 (5 μM), rPoCSF3-1M (5 μM), rTrx (5 μM), or PBS at 22°C for 2 h. After incubation, the bacteria were mixed with flounder serum, heat-inactivated flounder serum, or PBS at 22°C for 1 h. The mixture was then diluted, and the dilutions were plated on LB agar plates. The plates were incubated at 28°C for 48 h, and the colonies on the plates were counted. The survival rate was calculated as follows: (number of bacteria surviving serum treatment/number of bacteria surviving PBS treatment) × 100%.

### Statistical Analysis

All experiments were performed three times, and statistical analyses were carried out with GraphPad Prism 7 (GraphPad Software, USA) and SPSS 17.0 software (SPSS Inc., Chicago, USA). Data were analyzed with two-tailed student’s t-test and one-way analysis of variance (ANOVA), and a *p* value of less than 0.05 was considered statistically significant.

## Results

### PoCSF3-1 Is a Target Gene of pol-miR-novel_642

**Figure 1:**
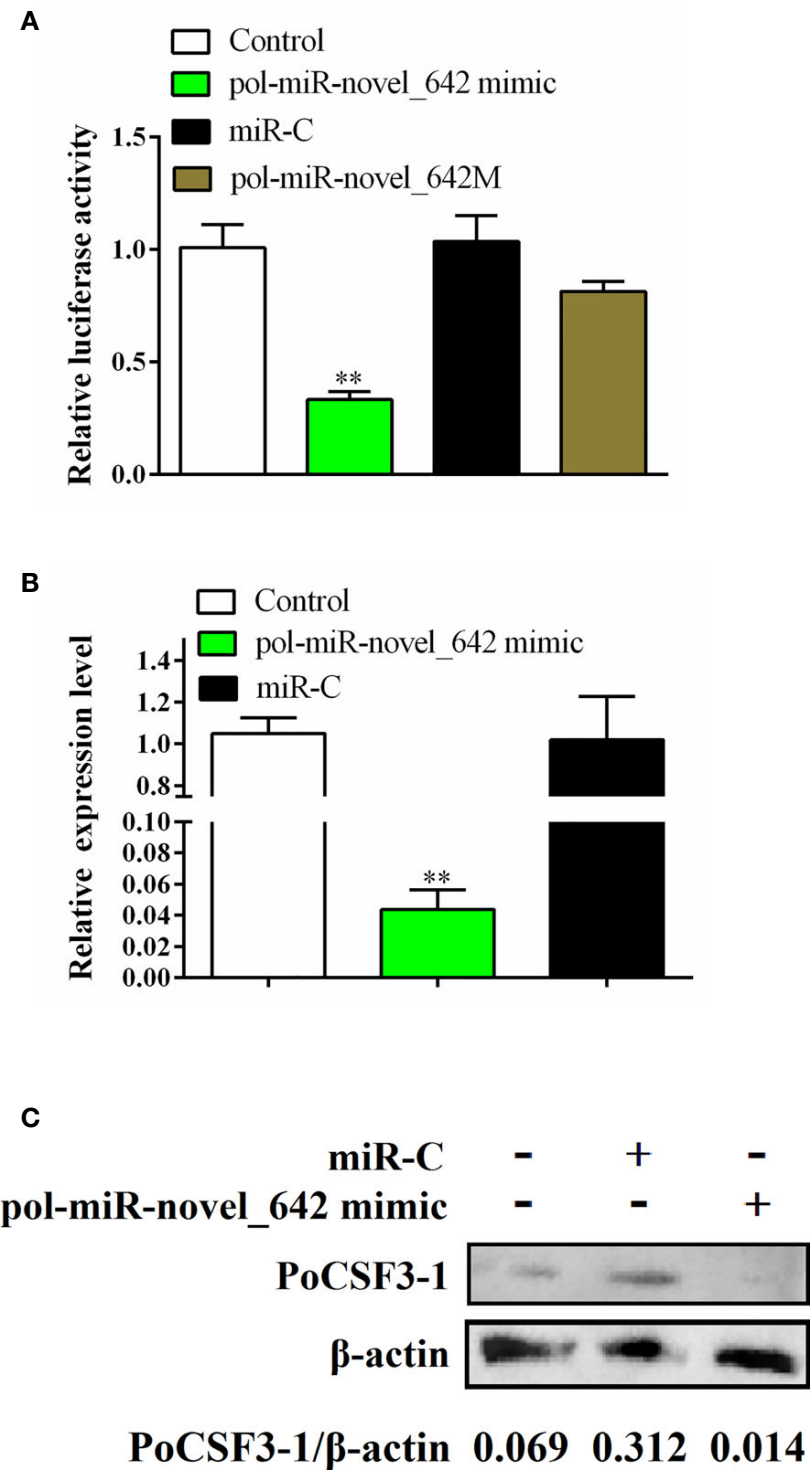
Regulation of PoCSF3-1 expression by pol-miR-novel_642. **(A)** HEK293T cells were transfected with pPoCSF3-1-Report in the absence (control) or presence of pol-miR-novel_642 mimic, miR-C (miRNA control), or pol-miR-novel_642M (mutant of pol-miR-novel_642) for 24 h, and the luciferase activity was then determined. **(B, C)** FG-9307 cells were transfected with or without (control) pol-miR-novel_642 mimic or miR-C, and PoCSF3-1 expression was determined by qRT-PCR **(B)** or Western blot with β-actin as a loading control **(C)**. In **(C)**, the relative densities of PoCSF3-1/β-actin are shown at the bottom of the figure. In **(A, B)**, values are the means of triplicate experiments and shown as means ± SEM. **p < 0.01.

Pol-miR-novel_642 was identified in a previous RNA sequencing study (11) as a novel miRNA with unknown function. Based on its seed sequence, pol-miR-novel_642 was predicted to target a 7-base region in the CDS of a gene encoding a hypothetical protein annotated as “uncharacterized protein LOC109626803 isoform X2” in the flounder genome (accession no. XP_019938527). XP_019938527 contains the conserved domain of IL6/G-CSF family and shares 62.87%-85.79% identities with the putative CSF3 of a number of fish, but only 35.35% identity with the known G-CSF of flounder (30, 38) (**Figure S2**). Structure modeling showed that XP_019938527 resembles highly mouse CSF3 in the overall structure (see *Recombinant PoCSF3-1 (rPoCSF3-1) Interacts With Bacteria in a Structure-Dependent Manner and Affects Bacterial Survival in Host Serum*). Based on these sequence features, this uncharacterized protein was named *Paralichthys olivaceus* CSF3-1 (PoCSF3-1). Luciferase reporter assay showed that pol-miR-novel_642 mimic significantly reduced the expression of the luciferase gene fused to PoCSF3-1 (**Figure 1A**). In contrast, a pol-miR-novel_642 variant, pol-miR-novel_642M, which bears mutation at the seed sequence involved in interaction with PoCSF3-1, did not affect the luciferase activity, suggesting that pol-miR-novel_642 modulated PoCSF3-1 expression in a manner that depended on the specific interaction between pol-miR-novel_642 and PoCSF3-1. Concordantly, when flounder FG-9307 cells were transfected with pol-miR-novel_642 mimic, PoCSF3-1 expression was significantly suppressed at both mRNA and protein levels (**Figures 1B, C**). These results indicate that PoCSF3-1 is a target gene of pol-miR-novel_642.

### PoCSF3-1 and pol-miR-novel_642 Expressions Are Regulated by Bacterial and Viral Pathogens

**Figure 2:**
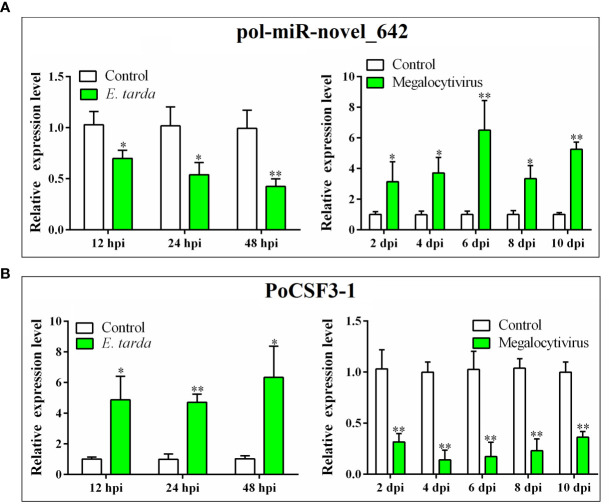
Expression of pol-miR-novel_642 **(A)** and PoCSF3-1 **(B)** in Japanese flounder in response to bacterial and viral infection. Flounder were infected with or without (control) *Edwardsiella tarda* or megalocytivirus. Pol-miR-novel_642 **(A)** and PoCSF3-1 **(B)** expression in kidney was determined by qRT-PCR at different hour post-infection (hpi) or day post-infection (dpi). Values are the means of triplicate experiments and shown as means ± SD. *p < 0.05; **p < 0.01.

*In vivo* study showed that the expressions of PoCSF3-1 and pol-miR-novel_642 in flounder were regulated by bacterial (*E. tarda*) and viral (megalocytivirus) pathogens (**Figure 2**). During *E. tarda* infection, pol-miR-novel_642 was significantly downregulated at 12, 24, and 48 hpi, whereas PoCSF3-1 expression was significantly upregulated at these time points (**Figure 2**). During megalocytivirus infection, pol-miR-novel_642 was significantly upregulated at 2, 4, 6, 8, and 10 dpi, whereas PoCSF3-1 expression was significantly downregulated at these time points (**Figure 2**).

### PoCSF3-1 and pol-miR-novel_642 Affect Pathogen Infection

**Figure 3:**
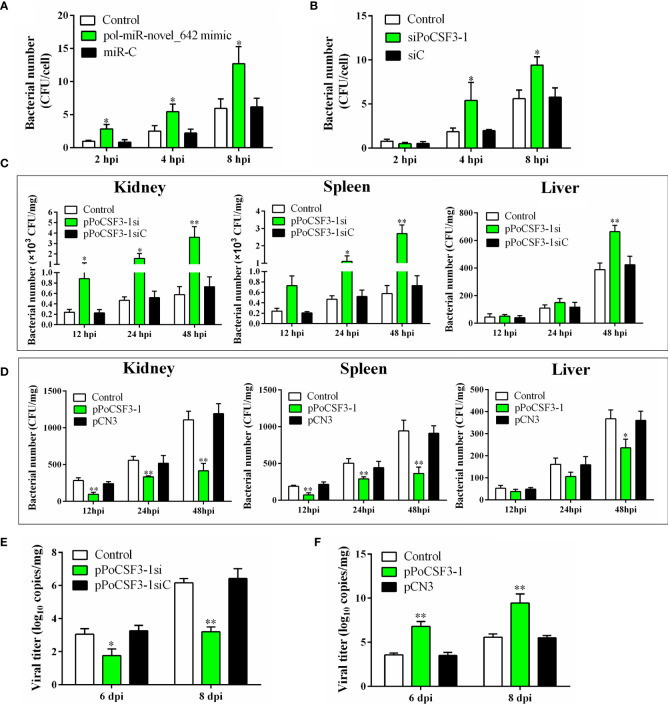
*In vitro* and *in vivo* effects of pol-miR-novel_642 and PoCSF3-1 on pathogen infection. **(A)** FG-9307 cells were transfected with or without (control) pol-miR-novel_642 mimic or miR-C (control miRNA) and then infected with *Edwardsiella tarda*. Intracellular bacterial number was determined at different hours post infection (hpi) and shown as CFU (colony forming unit). **(B)** FG-9307 cells were transfected with or without (control) siPoCSF3-1 or siC (control siRNA) and then infected with *E. tarda*. Intracellular bacterial number was determined as above. **(C)** Flounder were infected with *E. tarda* in the presence or absence (control) of pPoCSF3-1si or pPoCSF3-1siC (control plasmid). Bacterial loads in kidney, spleen, and liver were determined at different hpi. **(D)** Flounder were infected with *E. tarda* in the presence or absence (control) of pPoCSF3-1 or the control plasmid pCN3. Bacterial loads in tissues were determined as above. **(E)** Flounder were infected with megalocytivirus in the presence or absence (control) of pPoCSF3-1si or pPoCSF3-1siC, and viral copies in kidney were determined at 6 and 8 days post-injection (dpi). **(F)** Flounder were infected with megalocytivirus in the presence or absence (control) of pPoCSF3-1 or pCN3, and viral copies in kidney were determined as above. In all panels, values are the means of triplicate experiments and shown as means ± SD. *p < 0.05; **p < 0.01.

When flounder FG-9307 cells were transfected with siPoCSF3-1 (a siRNA targeting PoCSF3-1) that interferes with PoCSF3-1 expression, or when FG-9307 cells were transfected with pol-miR-novel_642 mimic, *E. tarda* infection in the cells was significantly enhanced (**Figures 3A, B**). Consistently, *in vivo* study showed that knockdown of PoCSF3-1 in flounder by administration of the plasmid pPoCSF3-1si (expressing a PoCSF3-1-targeting siRNA) significantly promoted *E. tarda* dissemination in fish tissues, whereas overexpression of PoCSF3-1 in flounder by administration of the plasmid pPoCSF3-1 (expressing PoCSF3-1) significantly inhibited *E. tarda* dissemination (**Figures 3C, D**). In addition to *E. tarda*, PoCSF3-1 also influenced megalocytivirus infection. PoCSF3-1 knockdown and overexpression significantly inhibited and promoted, respectively, megalocytivirus infection in flounder at 6 and 8 dpi (**Figures 3E, F**).

### PoCSF3-1 and pol-miR-novel_642 Regulate Autophagy

**Figure 4:**
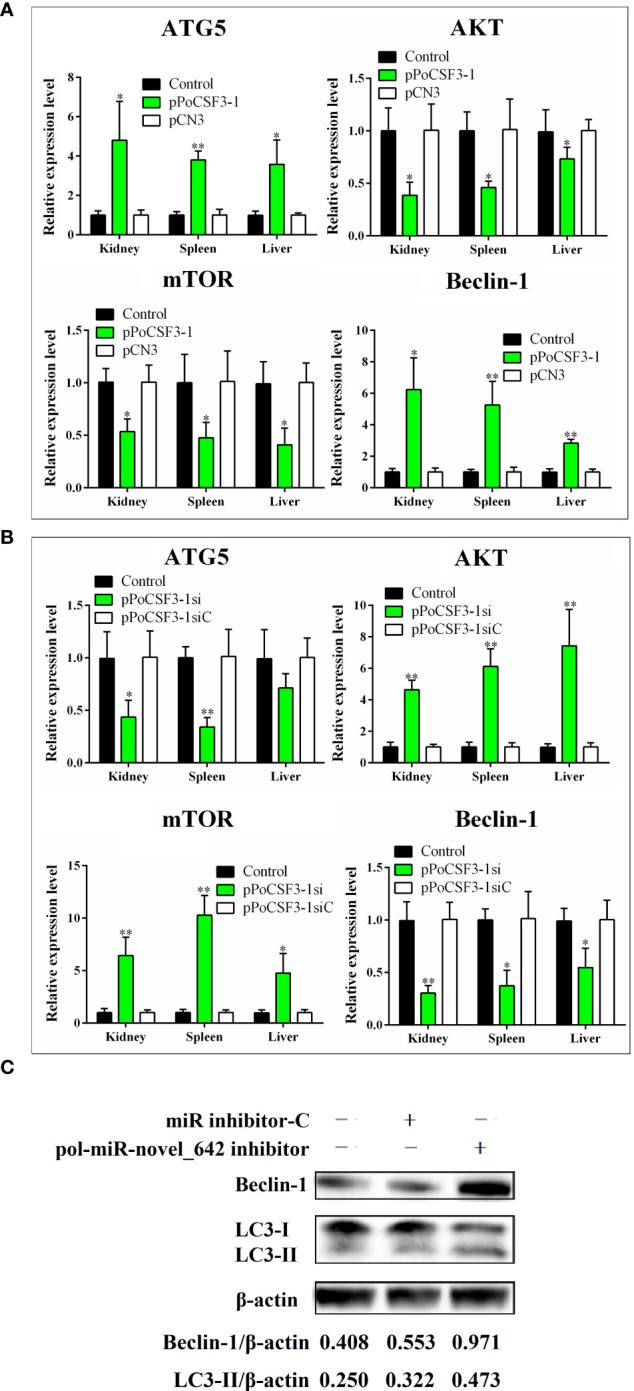
Effects of pol-miR-novel_642 and PoCSF3-1 on autophagy. **(A)** Flounder were administered with pPoCSF3-1, pCN3 (control plasmid), or PBS (control) for 7 days. The expressions of ATG5, AKT, mTOR, and beclin-1 in kidney, spleen and liver were determined by qRT-PCR. **(B)** Flounder were administered with pPoCSF3-1si, pPoCSF3-1siC (control plasmid), or PBS (control) for 7 days. Gene expression was determined as above. **(C)** FG-9307 cells were transfected with or without (control) pol-miR-novel_642 inhibitor or miR inhibitor-C (inhibitor control) for 24 h Beclin-1 and LC3-II were detected by Western blot. β-actin was used as a loading control. The relative densities of beclin-1/β-actin and LC3-II/β-actin are shown at the bottom of the figure. For panels **(A, B)**, values are the means of triplicate experiments and shown as means ± SD. *p < 0.05; **p < 0.01.

The mechanism behind the obseved effect of PoCSF3-1 on pathogen infection was explored. qRT-PCR showed that in flounder with PoCSF3-1 overexpression, the expressions of AKT and mTOR, which are known to inhibit autophagy in mammals, were significantly repressed in kidney, spleen, and liver, while the expressions of two other autophagy related genes, ATG5 and beclin-1, were significantly induced (**Figure 4A**). The reverse expression patterns were detected in flounder with PoCSF3-1 knockdown (**Figure 4B**). Consistently, in FG-9307 cells transfected with pol-miR-novel_642 inhibitor, which inhibited pol–miR-novel_642 expression, augmented production of beclin-1 and LC3-II were detected (**Figure 4C**), suggesting activation of autophagy.

### Recombinant PoCSF3-1 (rPoCSF3-1) Interacts With Bacteria in a Structure-Dependent Manner and Affects Bacterial Survival in Host Serum

**Figure 5:**
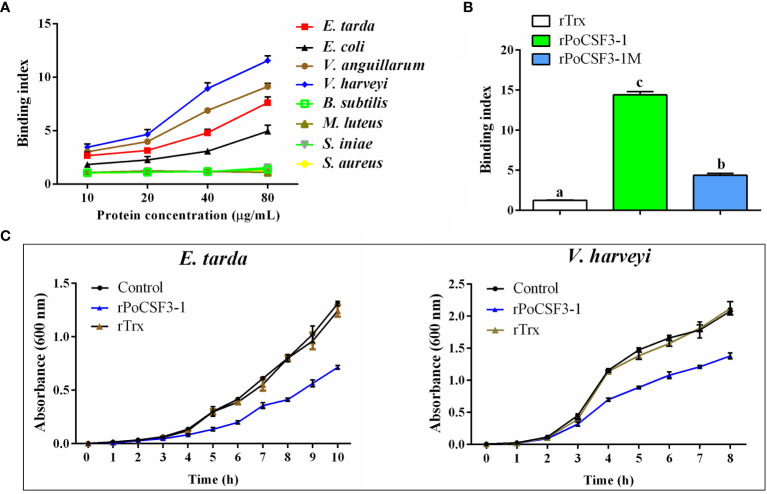
Interaction of rPoCSF3-1 variants with bacteria and its effect on bacterial growth. **(A)** Edwardsiella tarda, Escherichia coli, Vibrio anguillarum, Vibrio harveyi, Bacillus subtilis, Micrococcus luteus, Streptococcus iniae, and Staphylococcus aureus were incubated with or without (control) different concentrations of rPoCSF3-1 for 2 h The bound rPoCSF3-1 was determined by ELISA. **(B)** Lipid A was incubated with rPoCSF3-1, rPoCSF3-1M, rTrx, or PBS (control) for 2 h, and the protein-bound lipid A was determined by ELISA. Different letters indicate statistical significance (p < 0.05). **(C)** *E. tarda* and V. harveyi were incubated with or without (control) rPoCSF3-1 or rTrx, and bacterial growth was monitored at different hours. Values are the means of triplicate experiments and shown as means ± SEM. Different letters indicate statistical significance (p < 0.05).

**Figure 6:**
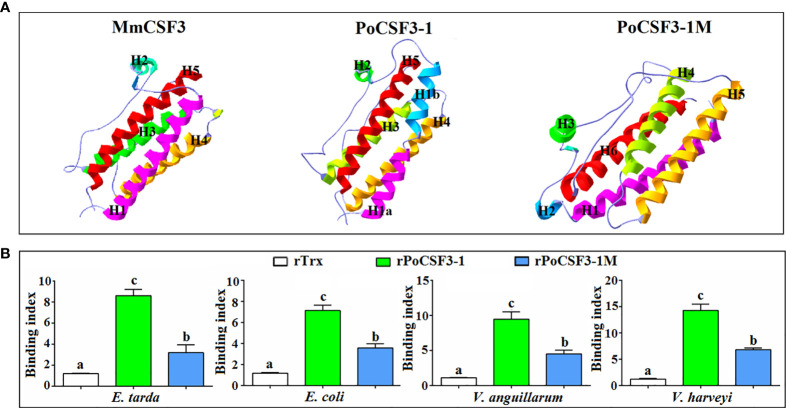
Structural modeling of PoCSF3-1 **(A)** and the structure-dependent binding of PoCSF3-1 to bacteria **(B)**. **(A)** The overall three-dimensional structures of PoCSF3-1 and PoCSF3-1M were modeled using SWISS-MODEL based on the template protein of mouse CSF3 (PDB: 1PGR) and shown in ribbons with rainbow colors. **(B)** Bacteria were incubated with rPoCSF3-1, rPoCSF3-1M, rTrx, or PBS (control) for 2 h, and the bacteria-bound protein was determined by ELISA. Values are the means of triplicate experiments and shown as means ± SEM. Different letters indicate statistical significance (*p* < 0.05).

His-tagged rPoCSF3-1 was purified (**Figure S3**). ELISA showed that rPoCSF3-1 was able to bind Gram-negative bacteria, including *E. tarda*, *Escherichia coli*, *Vibrio anguillarum*, and *Vibrio harveyi*, but did not bind Gram-positive bacteria (**Figure 5A**). Consistently, rPoCSF3-1 exhibited apparent binding to lipid A (**Figure 5B**). In contrast, no bacteria binding activity was detected with mouse CSF3 (data not shown). rPoCSF3-1 did not kill the bound bacteria, but inhibited the growth of some of the bacteria, i.e., *E. tarda* and *V. harveyi* (**Figure 5C**). Structure modeling showed that both PoCSF3-1 and mouse CSF3 contain predominantly α–helixes; however, the H1 α–helix in mouse CSF3 is replaced largely by two smaller α–helixes (H1a and H1b) in PoCSF3-1 (**Figure 6A**). The difference is likely contributed primarily by the motif of ^36^ILL^39^ in PoCSF3-1, as mutation of this motif to ^36^SLE^39^ resulted in the disappearance of the two small α–helixes and the emergence of a large α–helix similar to that of mouse H1 (**Figure 6A**). Compared to the wild type rPoCSF3-1, the mutant rPoCSF3-1M that bears the ^36^SLE^39^ substitution exhibited significantly reduced binding to bacteria (**Figure 6B**). Serum survival analysis showed that the presence of rPoCSF3-1, but not rPoCSF3-1M, significantly enhanced the survival rate of *E. tarda* in flounder serum to the level of 100% (**Figure S4**). In contrast, rPoCSF3-1 had no effect on *E. tarda* survival in heat-inactivated serum (data not shown).

### rPoCSF3-1 Regulates the Activation and Bacterial Resistance of HKLs

**Figure 7:**
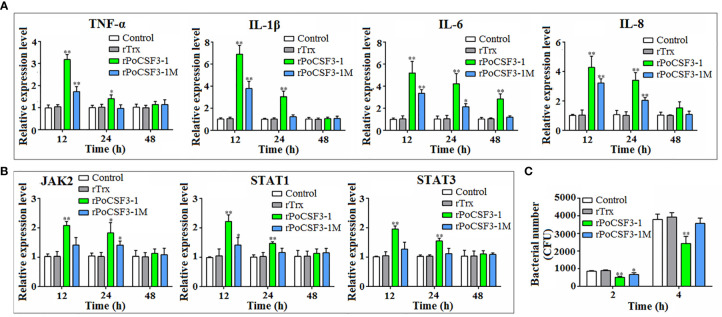
Effects of rPoCSF3-1 on immune gene expression and bacterial infection in head kidney leukocytes (HKLs). **(A, B)** Flounder HKLs were incubated with or without (control) rPoCSF3-1, rPoCSF3-1M, or rTrx for different hours. The expressions of the immune genes were determined by qRT-PCR. **(C)** Flounder HKLs were infected with *Edwardsiella tarda* in the presence or absence (control) of rPoCSF3-1, rPoCSF3-1M, or rTrx. Intracellular bacterial numbers (shown as colony forming unit, CFU) were determined at 2 and 4 hpi. Values are the means of triplicate experiments and shown as means ± SEM. *p < 0.05; **p < 0.01.

When HKLs were treated with rPoCSF3-1, the cells exhibited significantly enhanced proliferation (**Figure S5**). In these cells, the expression levels of the inflammatory cytokines of TNF-α, IL-1β, IL-6, and IL-8 significantly increased at 12 h and 24 h post-treatment (**Figure 7A**). The mutant rPoCSF3-1M also enhanced the expression of the cytokines, but to significantly lower degrees than that caused by rPoCSF3-1. Similarly, rPoCSF3-1 significantly upregulated the expression of the JAK/STAT pathway genes JAK2, STAT1, and STAT3 at 12 h and 24 h post-treatment, while rPoCSF3-1M only moderately, though significantly, upregulated the expression of JAK2 and STAT1, but not the expression of STAT3 (**Figure 7B**). Cellular infection showed that when HKLs were infected with *E. tarda* in the presence of rPoCSF3-1, the intracellular bacterial loads were significantly reduced compared to that in the control cells or in rPoCSF3-1M-treated cells (**Figure 7C**).

## Discussion

Accumulating evidences have demonstrated that pathogen infection can induce extensive host miRNA response, which provides a potential mechanism for host antibacterial defense and pathogen manipulation of the host system (54–56). In this study, we found that flounder pol-miR-novel_642, a novel miRNA with unknown function, exhibited differential expression during *E. tarda* and megalocytivirus infection, suggesting an involvement of pol-miR-novel_642 in flounder immunity against bacterial and viral pathogens. In animals, miRNAs decrease mRNA stability or inhibit mRNA translation mostly through binding the 3’UTR of the target genes (4, 5, 57), while in plants, miRNA targets are often present in the coding regions of the target genes (58, 59). However, recent studies demonstrated that the coding regions of animal genes, such as zebrafish let-7 and human DNA methyltransferase 3b, can be targeted by miRNAs (1, 6, 8). In our study, luciferase reporter assay showed that pol-miR-novel_642 negatively regulated a hypothetical gene encoding an uncharacterized protein, which was named PoCSF3-1 in this study based on its structural similarity with mammalian CSF3, by specific interaction with the coding region of PoCSF3-1. Consistently, overexpression of pol-miR-novel_642 in flounder cells effectively decreased the mRNA and protein levels of PoCSF3-1. These results indicate that PoCSF3-1 is a target gene of pol-miR-novel-642.

The antimicrobial effect of CSF3/G-CSF has been widely reported. In humans, G-CSF was induced by human respiratory syncytial virus (hRSV) and constituted an important modulator of neutrophil infiltration during hRSV infection (60). In mice, G-CSF expression was elevated upon uropathogenic *E. coli* infection, and G-CSF-induced emigration of neutrophils was thought to be critical for bacterial clearance (61). G-CSF was required for the induction of circulating neutrophils elicited by *L. monocytogenes* and suppression of bacterial invasion in mouse tissues (28). In fish, the G-CSF of rock bream was highly expressed after *S. iniae* and *E. tarda* challenge (33), and the G-CSF of Japanese flounder, which shares 35.35% identity with the PoCSF3-1 identified in this study, showed altered expression during pathogen infection and regulated immune gene expression and bacterial infection (30, 38). In this study, PoCSF3-1 expression was modulated by *E. tarda* and megalocytivirus in a way opposite to that of pol-miR-novel_642 expression, which supported the negative regulatory relationship between pol-miR-novel_642 and PoCSF3-1. PoCSF3-1 knockdown or pol-miR-novel_642 overexpression significantly augmented *E. tarda* infection, whereas PoCSF3-1 overexpression significantly attenuated *E. tarda* infection. These observations indicate a negative effect of PoCSF3-1 on *E. tarda* infection. Since PoCSF3-1 expression was upregulated by *E. tarda*, it is likely that during the infection process, flounder activates PoCSF3-1 expression as a defense mechanism to eliminate the invading *E. tarda*. In contrast, PoCSF3-1 overexpression promoted megalocytivirus infection, whereas PoCSF3-1 knockdown suppressed megalocytivirus infection, suggesting that PoCSF3-1 facilitated megalocytivirus infection. Since PoCSF3-1 expression was downregulated from the early to late stages of megalocytivirus infection, it is likely that during the infection, flounder reduces the expression level of PoCSF3-1 as a strategy to block the viral infection. Together, these results indicate that PoCSF3-1 is involved in flounder immune defense against microbial pathogens, and that PoCSF3-1 plays different roles in *E. tarda* and megalocytivirus infection, which may be due to the different infection mechanisms of the bacterial and viral pathogens. qRT-PCR showed that PoCSF3-1 overexpression upregulated the expression of ATG5 and beclin-1, which promote autophagy (62, 63), and downregulated the expression of AKT and mTOR, which inhibited autophagy (64, 65). Consistently, pol-miR-novel_642 knockdown enhanced the production of beclin 1 and LC3 II. These results indicate that, like human CFS3 that is known to induce autophagy in an acute spinal cord injury model (66), PoCSF3-1 is able to activate the process of autophagy, which probably contributes to the observed effects of PoCSF3-1 on pathogen infection.

G-CSF has long been known as a cytokine, and no reports on direct interaction between G-CSF and bacteria have been documented. In our study, rPoCSF3-1 was found to bind Gram-negative bacteria, probably *via* interaction with lipid A. Binding of rPoCSF3-1 markedly inhibited the growth of *E. tarda*, which may explain, at least in part, the *in vivo* observation that PoCSF3-1 attenuated *E. tarda* infection. Unlike PoCSF3-1, the mutant protein rPoCSF3-1M, which bears the ^36^ILL^39^ to ^36^SLE^39^ substitution, displayed a significantly impaired ability to bind bacteria and lipid A. Structural modeling showed that mutation of ^36^ILL^39^ to ^36^SLE^39^ resulted in the loss of the PoCSF3-1-characteristic α–helixes and enabled the mutant resembling more the mouse CSF3. These results suggest that the α–helixes involving ^36^ILL^39^ are essential to bacterial interaction, and the lack of this structural feature in mouse CSF3 may possibly account for the inability of mouse CSF3 to bind bacteria.

In mammals, G-CSF is a key modulator responsible for the proliferation of neutrophils, endothelial cells, and macrophages (24, 67). Mammalian G-CSF is known to activate the JAK/STAT pathway (68, 69). For example, human G-CSF promoted phosphorylation of JAK1, JAK2, STAT3 and STAT5 in placenta or neutrophils (70, 71). In teleost, large yellow croaker GCSFa could promote the proliferation of primary head kidney leucocytes (PKL) and elevate the expression of STAT3 and STAT5 (39). In this study, we found that rPoCSF3-1 stimulated the proliferation of HKLs and enhanced the expression of inflammatory cytokines and JAK2, STAT1, and STAT3, indicating an immune regulatory function of rPoCSF3-1 involving specific JAK/STAT pathways. Consistently, rPoCSF3-1 increased the ability of HKLs to resist *E. tarda* infection, which is likely due to the augmented immune response induced by rPoCSF3-1. It is interesting that compared to rPoCSF3-1, rPoCSF3-1M exhibited much weaker effects on immune gene expression and bacterial infection, suggesting further the importance of the ^36^ILL^39^ motif in the activity of PoCSF3-1.

In conclusion, in this study we unraveled the functions of the novel miRNA pol-miR-novel_642 and its target gene PoCSF3-1. Our results indicate that pol-miR-novel_642 and PoCSF3-1 play a critical role in pathogen infection by affecting host immune response including autophagy, inflammation, and cellular proliferation. Importantly, we demonstrated that rPoCSF3-1 possesses the ability to directly interact with and inhibit the growth of some bacteria, and both the bacterial interaction and the immune effect of rPoCSF3-1 depend on a characteristic structural motif that is absent in murine CSF3. These results add new insights into the function of flounder CSF3 and suggest a diversified role of fish CSF3 as both a cytokine and a bacterial binding protein.

## Data Availability Statement

The datasets presented in this study can be found in online repositories. The names of the repository/repositories and accession number(s) can be found in the article/**Supplementary Material**.

## Ethics Statement

The animal study was reviewed and approved by The Ethics Committee of the Institute of Oceanology, Chinese Academy of Sciences.

## Author Contributions

LS conceived the study. WL conducted the experiments. BS prepared the HKLs. WL, BS, and XG analyzed the data. WL wrote the first draft of the manuscript. LS edited the manuscript. All authors contributed to the article and approved the submitted version.

## Funding

This work was supported by the grants of the National Natural Science Foundation of China (31730100), the Marine S&T Fund of Shandong Province for Pilot National Laboratory for Marine Science and Technology (Qingdao) (2018SDKJ0302-2), and the Taishan Scholar Program of Shandong Province.

## Conflict of Interest

The authors declare that the research was conducted in the absence of any commercial or financial relationships that could be construed as a potential conflict of interest.

## Publisher’s Note

All claims expressed in this article are solely those of the authors and do not necessarily represent those of their affiliated organizations, or those of the publisher, the editors and the reviewers. Any product that may be evaluated in this article, or claim that may be made by its manufacturer, is not guaranteed or endorsed by the publisher.
