# Supplementary material for: A Novel microRNA of Japanese Flounder Regulates Antimicrobial Immunity Involving a Bacteria-Binding CSF3
Source: Front Immunol. 2021 Aug 19;12:723401. doi: 10.3389/fimmu.2021.723401 (PMC8417112; doi:10.3389/fimmu.2021.723401)
Supplement: Supplementary file 1 [file DataSheet_1.docx]

**Supplementary data**

**Table S1. Primers used in this study.**

| **Primer** | **Sequence (5'→3')** **^a^** |
| --- | --- |
| pol-miR-novel_642-F | GCGAAGGCTGCTGCACTC |
| pol-miR-novel_642-R | AGTGCAGGGTCCGAGGTATT |
| PoCSF3-1-F | CACACGGAGACTCTGCAACT |
| PoCSF3-1-R | CCTGAACCTCGTATTCCCCG |
| PoCSF3-1-CDS-F1 | TGTTTAAACGAGCTCGCTAGCATGGCCACGCTCGCCCGC (Nhe I) |
| PoCSF3-1-CDS-R1 | CTTGCATGCCTGCAGGTCGACTCAGCTCTCCATCTCCTC (Sal I) |
| PoCSF3-1-CDS-F2 | GGCTAGCCTCGAGATATCATGGCCACGCTCGCCCGC (EcoR V) |
| PoCSF3-1-CDS-R2 | TCCTCCTCCTCCTCCGATATCTCAGCTCTCCATCTCCTC (EcoR V) |
| PoCSF3-1-CDS-F3 | ATGGGTCGCGGATCCGAATTCATGGCCACGCTCGCCCGC (EcoR I) |
| PoCSF3-1-CDS-R3 | CTCGAGTGCGGCCGCAAGCTTTCAGCTCTCCATCTCCTC (Hind III) |
| PoCSF3-1-Mut-F | CCGGAGCCGCAGCCTGGCGCACAAGAGCCTGGAATCCGTGCCCGACGCG |
| PoCSF3-1-Mut-R | TGCACCAGCTCCTGGGGCCTCGGCGTCGGACCGCGTGTTCTTCCAGGCT |
| ATG5-F | AACAGCTGTGGATGGGTCTG |
| ATG5-R | ACAGGGCGAAACAGTCTCTG |
| AKT-F | AGCTGATGAAGACGGAACGG |
| AKT-R | GGGTCTGGAGAGGAGTCCAT |
| mTOR-F | TCTTCCACAATCGCCAGCTT |
| mTOR-R | GAGCCAGTCGTCTTTGGACA |
| Beclin-1-F | TCAATGTGCTGGATCGGGTC |
| Beclin-1-R  TNF-α-F  TNF-α-R  IL-1β-F  IL-1β-R  IL-6-F  IL-6-R  IL-8-F  IL-8-R  JAK2-F  JAK2-R  STAT1-F  STAT1-R  STAT3-F  STAT3-R | GCGCTCTCTGTGGACATCAT  TACAGCCAGGCGTCATTCAG  GCCCAGGTAGATGGCATTGTA  CAGCACATCAGAGCAAGACAACA  TGGTAGCACCGGGCATTCT  CTCCAGTCGAATACGAGCCC  ACTCTTTCTGGTGGTGAGCG  GCCTGAGAAGCCTAGGAGTG  TGACTCTCTTCACCCACGGA  ATCTGCTGGGGGACAAAGTG  CCCCGACATTATCACCAGGG  GTACTACCCCAAACCCGTGG  TTGAAAGTCTGCTCTCATCACA  CATCCCCAAAGAGGAAGCGT  GTCCATGAACACGGAGGGAC |
| 5s rRNA-F | CCATACCACCCTGAACAC |
| 5s rRNA-R | CGGTCTCCCATCCAAGTA |
| ^a^Underlined nucleotides are restriction sites. | |

**Figure S1**. Verification of PoCSF3-1 knockdown (A) and overexpression (B) in flounder. (A) Flounder were administered with pPoCSF3-1, pCN3, or PBS (control). (B) Flounder were administered with pPoCSF3-1si, pPoCSF3-1siC, or PBS (control). In both panels, PoCSF3-1 expression in kidney, spleen, and liver was determined by qRT-PCR at 7 d post-plasmid injection. The expression level of the control fish was set as 1. Values are the means of triplicate experiments and shown as means ± SD. **p* < 0.05; ***p* < 0.01.


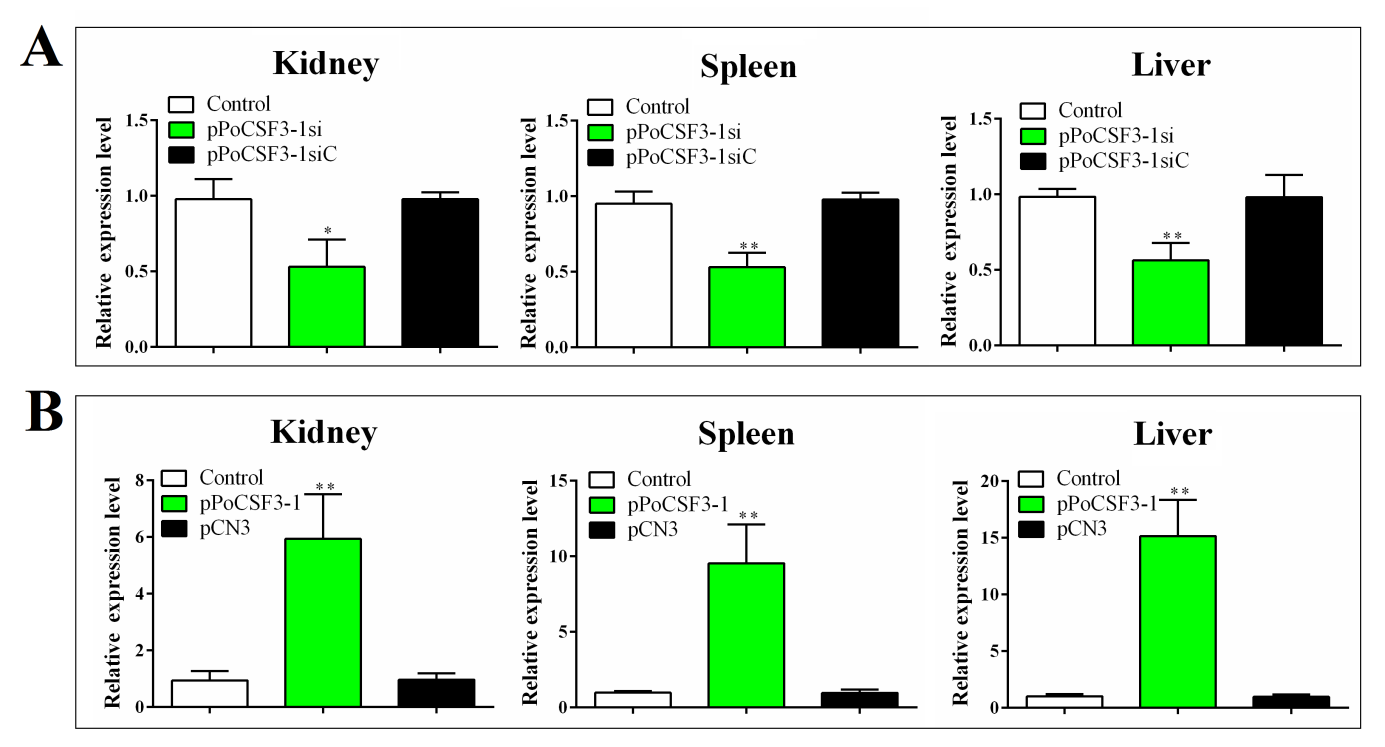


**Figure S2.** Alignment of the sequences of PoCSF3-1 homologues. Dots denote gaps introduced for maximum matching. The consensus residues are in blue, the residues that are ≥75% identical among the aligned sequences are in pink. The GenBank accession numbers of the aligned sequences are as follows. *Hippoglossus stenolepis*, XP_035001744.1; *Sander lucioperca*, XP_031147046.1; *Anabas testudineus*, XP_026206534.1; *Mastacembelus armatus*, XP_026171630.1; *Amphiprion ocellaris*, XP_035811372.1; *Cyclopterus lumpus*, XP_034414164.1; *Notolabrus celidotus*, XP_034563982.1; *Acanthopagrus latus*, XP_036937430.1; *Cynoglossus semilaevis*, XP_016889454.1; Flounder G-CSF, BAE16320.1; *Mus musculus*, EDL16160.1.


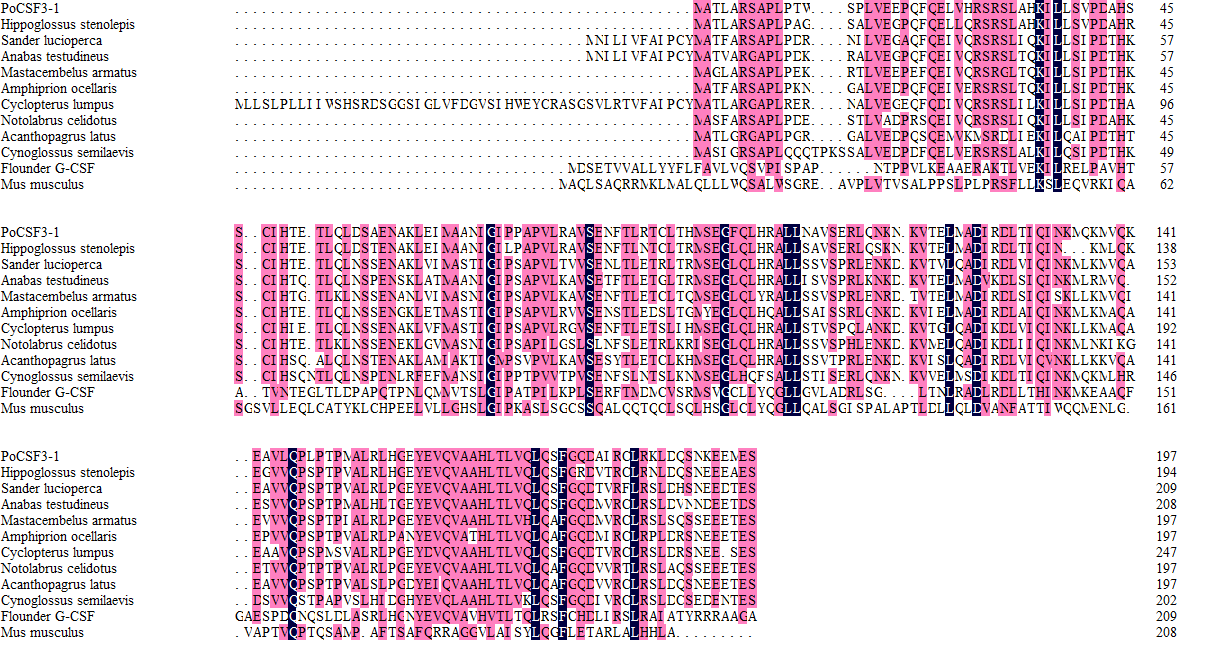


**Figure S3.** SDS-PAGE analysis of purified recombinant proteins. Purified rPoCSF3-1 (A, lane 2) and rPoCSF3-1M (B, lane 2) were analyzed by SDS-PAGE and viewed after staining with Coomassie brilliant blue R-250. Lane 1, protein markers.

**
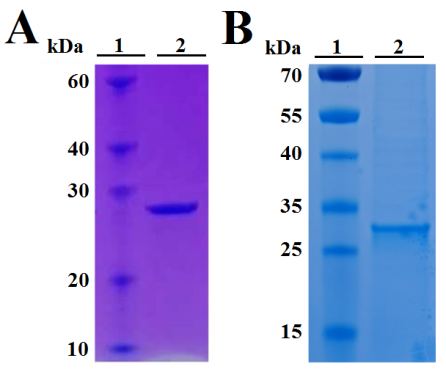
**

**Figure S4**. The effect of rPoCSF3-1 on the survival of *Edwardsiella tarda* in host serum. *E. tarda* was incubated with or without (control) rPoCSF3-1, rPoCSF3-1M, or rTrx for 2 h, and then incubated with or without (control) flounder serum for 1 h. The relative survival rate of the bacteria was determined after the incubation. Values are the means of triplicate experiments and shown as means ± SEM. ***p* < 0.01.

**

**

**Figure S5**. Effects of rPoCSF3-1 and rPoCSF3-1M on the proliferation of head kidney leukocytes (HKLs). Flounder HKLs were incubated with or without (control) rPoCSF3-1, rPoCSF3-1M, or rTrx for different hours. Cell proliferation was determined by measuring absorbance at 450 nm. Values are the means of triplicate experiments and shown as means ± SEM. **p* < 0.05; ***p* < 0.01.
